# Collodion as an Erector of Flat, Undeveloped Nipples

**Published:** 1858-04

**Authors:** 


### COLLODION AS AN ERECTOR OF FLAT, UNDEVELOPED NIPPLES.

By coating the vicinity of the nipple, in a circle of an inch and a half wide, with collodion, the compression effected during the contraction of this adhesive material, will cause the nipple to be protruded sufficiently to allow of convenient nursing when otherwise it would be entirely impracticable. There should be a space of half an inch of the areola around and near the nipple left uncovered by the collodion. It is well known that if the nipple can be drawn until the tumefaction of the mamma somewhat subsides, there will be no further trouble, and it is believed this simple means will often relieve us of much anxiety upon this score.—*Ibid.*

